# Using an integral projection model to assess the effect of temperature on the growth of gilthead seabream *Sparus aurata*

**DOI:** 10.1371/journal.pone.0196092

**Published:** 2018-05-03

**Authors:** F. J. Heather, D. Z. Childs, A. M. Darnaude, J. L. Blanchard

**Affiliations:** 1 Dept. Animal and Plant Sciences, University of Sheffield, Western Bank, Sheffield, United Kingdom; 2 MARBEC, CNRS-Univ. Montpellier-Ifremer-IRD (Montpellier)–Université de Montpellier, Place Eugène Bataillon, Montpellier, France; 3 Institute of Marine and Antarctic Studies, University of Tasmania, Castray Esplanade, Hobart, Australia; Institut National de la Recherche Agronomique, FRANCE

## Abstract

Accurate information on the growth rates of fish is crucial for fisheries stock assessment and management. Empirical life history parameters (von Bertalanffy growth) are widely fitted to cross-sectional size-at-age data sampled from fish populations. This method often assumes that environmental factors affecting growth remain constant over time. The current study utilized longitudinal life history information contained in otoliths from 412 juveniles and adults of gilthead seabream, *Sparus aurata*, a commercially important species fished and farmed throughout the Mediterranean. Historical annual growth rates over 11 consecutive years (2002–2012) in the Gulf of Lions (NW Mediterranean) were reconstructed to investigate the effect of temperature variations on the annual growth of this fish. *S*. *aurata* growth was modelled linearly as the relationship between otolith size at year *t* against otolith size at the previous year *t-1*. The effect of temperature on growth was modelled with linear mixed effects models and a simplified linear model to be implemented in a cohort Integral Projection Model (cIPM). The cIPM was used to project *S*. *aurata* growth, year to year, under different temperature scenarios. Our results determined current increasing summer temperatures to have a negative effect on *S*. *aurata* annual growth in the Gulf of Lions. They suggest that global warming already has and will further have a significant impact on *S*. *aurata* size-at-age, with important implications for age-structured stock assessments and reference points used in fisheries.

## Introduction

The gilthead seabream (*Sparus aurata* L.) is one of the main aquaculture fish species in Europe, with over 100,000 tonnes reared in 2012 [1]. It is found predominantly throughout the Mediterranean Sea, although it is also fished along the Eastern Atlantic coast of Great Britain to Senegal and in the Baltic Sea (rare) [2]. Whilst European wild captures of *S*. *aurata* have remained consistently fewer than 6,000 tonnes annually, aquaculture production of this species has increased exponentially since the 1990s and is now an order of magnitude greater than that of its landings [3]. Despite the commercial importance of this fish species, there appears to be limited information on the biology and ecology of its wild populations [3, 4], especially with regards to the environmental variables that control its growth [5]. Experimental studies have shown this species to be particularly sensitive to water temperature [6]. This sensitivity to water temperature could threaten wild *S*. *aurata* stocks in the future, especially in areas such as the Gulf of Lions (NW Mediterranean) where the species colonises shallow coastal lagoons each year to feed on and grow over the summer months [7, 8].

The Intergovernmental Panel on Climate Change suggest that, even with stringent mitigation measures to reduce carbon emissions in the following century, we can expect 1.0°C warming of mean global sea surface temperatures by the end of the century (Representation Concentration Pathway [RCP] 2.6) [9]. However, if we continue ‘business-as-usual’, ~3.0°C of sea surface temperature warming could occur (between RCP6.0 and RCP8.5), potentially resulting in severe consequences for biodiversity and the fishing economy [10].The Mediterranean Sea is believed to be one of the most impacted regions from climate change effects in the world [11, 12], and has been identified as one of the primary climate change ‘hot-spots’ [13]. Warming trends of ~1°C over the last three decades have already been observed in the shallows of the NW Mediterranean [11, 14], where *S*. *aurata* inhabits [2]. The consequences of this warming are already being observed, for example in species distributional shifts and extensions [15–17]. As has been shown for a wide range of ectotherms [18], increased temperature can affect fish growth and life-histories [19], potentially leading to reductions in adult size in temperate fish [20, 21].

For *S*. *aurata* the effects of local warming might be even more severe as anoxic bottom conditions in the shallow coastal lagoons have been observed following heat-wave events, such as that in the Thau lagoon in 2003 [22]. Anoxic bottom conditions can come about by high nitrogen and phosphorus concentrations in the summer, causing an algal bloom and subsequent increase of oxygen-consuming bacteria as dead algae sink to the bottom [23]. Reduced oxygen levels can potentially reduce fish growth directly through hypoxic stress [24] or indirectly through a reduction of food availability due to prey mortality [22, 25, 26]. However, reductions in size-at-age due to warming are not ubiquitous and responses to warming can vary substantially in their magnitudes or directions of change. Hence the detailed understanding of how environmental factors linked to temperature increase affect growth rates in wild fish populations is still needed to allow us to predict how ongoing global climate change may affect them.

Growth is an important component of population dynamics; affecting the size and age structure of populations through time. The von Bertalanffy growth function (VBGF) [27] is the most commonly applied growth function in fisheries science and is widely used to describe the asymptotic relationship between size and age for many fish species. The extensive use of this equation has resulted in the parameters being very useful in comparative studies and it has been widely implemented in stock assessment calculations [28].

Cross-sectional size-at-age information for fitting the VBGF has long been attained using solely fish body size and age at capture, with age often inferred from various time-keeping structures such as fish scales, vertebrae and otoliths [29]. Size and age at capture, however, only provide a snapshot in time of individual lifetime growth rates. Conversely, time-keeping structures such as otoliths offer a means to analyse the actual growth experienced by individuals throughout their life, avoiding the assumption that variables that affect growth rate remain constant over time [30]. This longitudinal growth data within individuals, such as the information contained within fish otoliths, might allow the development a more accurate estimation of fish population lifetime growth rate [31]. This could be particularly promising for S. aurata where the commonly applied VBGF, has been cautioned against in the case of wild populations for its low predictive power and where the need for multiple growth models have been emphasised [5].

In this study, we took advantage of the longitudinal growth data stored in otoliths to determine lifetime annual growth rates for 412 specimens of *S*. *aurata* (ages 1+) from the Gulf of Lions (NW Mediterranean), using otolith annual increment width as a proxy for individual-level growth within specified years [29, 30].

With each annual increment representing a given year of growth, it was then possible to associate the annual growth for each individual fish to the annual water temperatures that prevailed in the area. For this, we modelled growth linearly using the otolith size the previous year *t-1* to predict the otolith size the following year *t*, and used this linear model to parameterise a cohort integral projection model (cIPM) [32]; normally applied to terrestrial systems (although see Moore et al. [33]). This allowed us to estimate sizes-at-ages in the population and to make predictions about the individual-level growth rate change under different temperature scenarios.

## Materials and methods

### Study area

**Fig 1:**
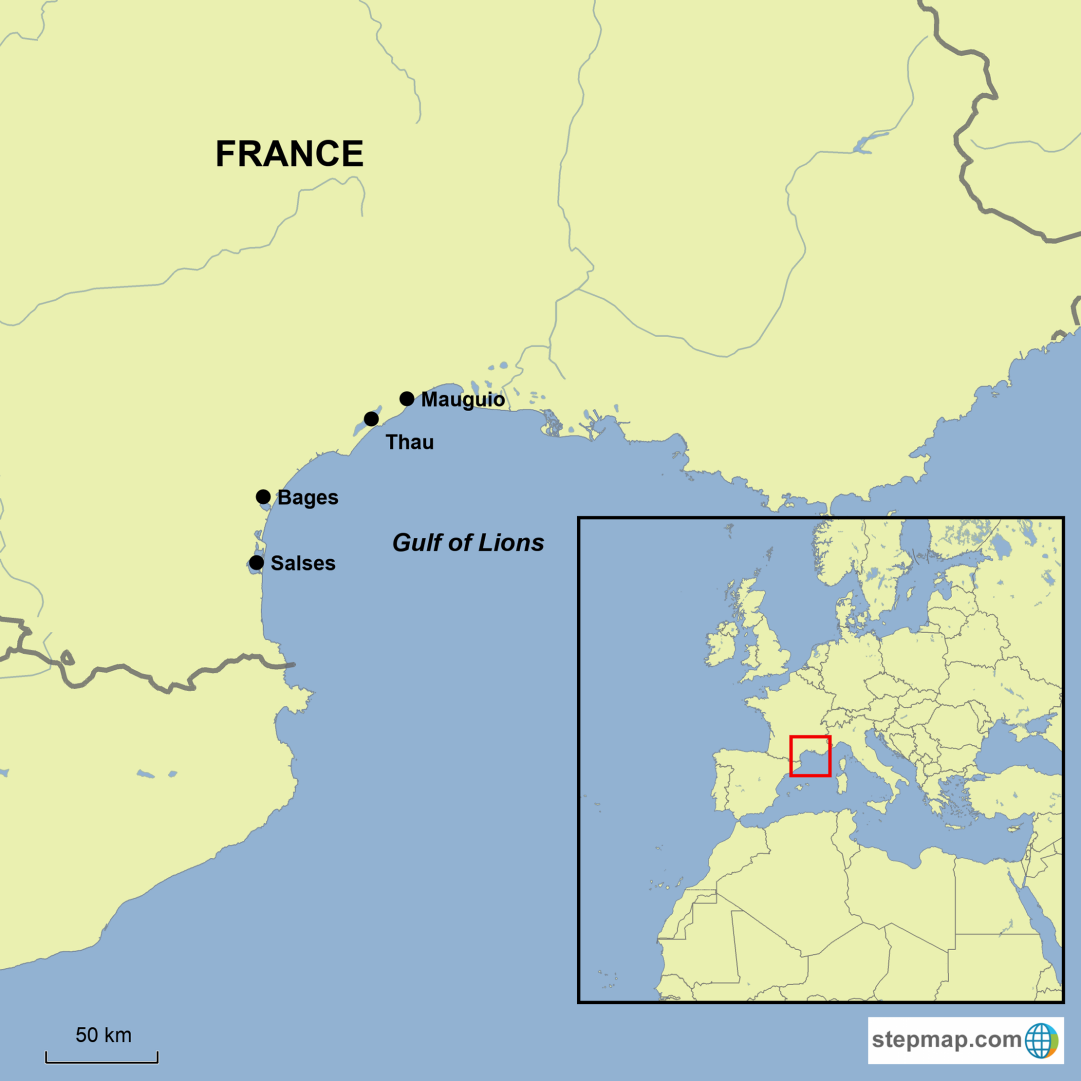
The study area. The Gulf of Lions (41.9–43.9°N, 2.6–5.9°E), NW Mediterranean Sea and associated four major coastal lagoons; Salses-Leucate (Salses), Bages-Sigean (Bages), Thau and Mauguio. This map was created using Stepmap online map editor [36].

The Gulf of Lions is situated in the north-western Mediterranean Sea, spanning 300km along the southern coastline of France (**Fig 1**). Running parallel to this embayment is a dense coastal lagoon system, comprising 17 lagoons spread along the coast and all connected to the sea. During the summer months, juvenile *S*. *aurata* migrate and occupy the coastal lagoons, migrating back out to sea in the autumn, when temperatures of the lagoons and open sea are similar [5]. This annual migration is repeated in the following years as an adult, although with reduced probability [5].

The Mauguio, Thau, Bages-Sigean (Bages) and Salses-Leucate (Salses) lagoons are the four largest lagoons in the system and provide important nurseries each year for *S*. *aurata*, among other commercially important fish species [3]. They differ in their chemical and hydrological properties, including temperature, salinity, pH, oxygenation and depth [34]. For this work, these four major lagoons were grouped into two shallow (Mauguio and Bages, with mean depths of 0.8 and 1.3m, respectively) and two deep lagoons (Salses and Thau, 2 and 4m, respectively), as lagoon depth significantly affects temperature and oxygen content [35].

### Fish otoliths

**Fig 2:**
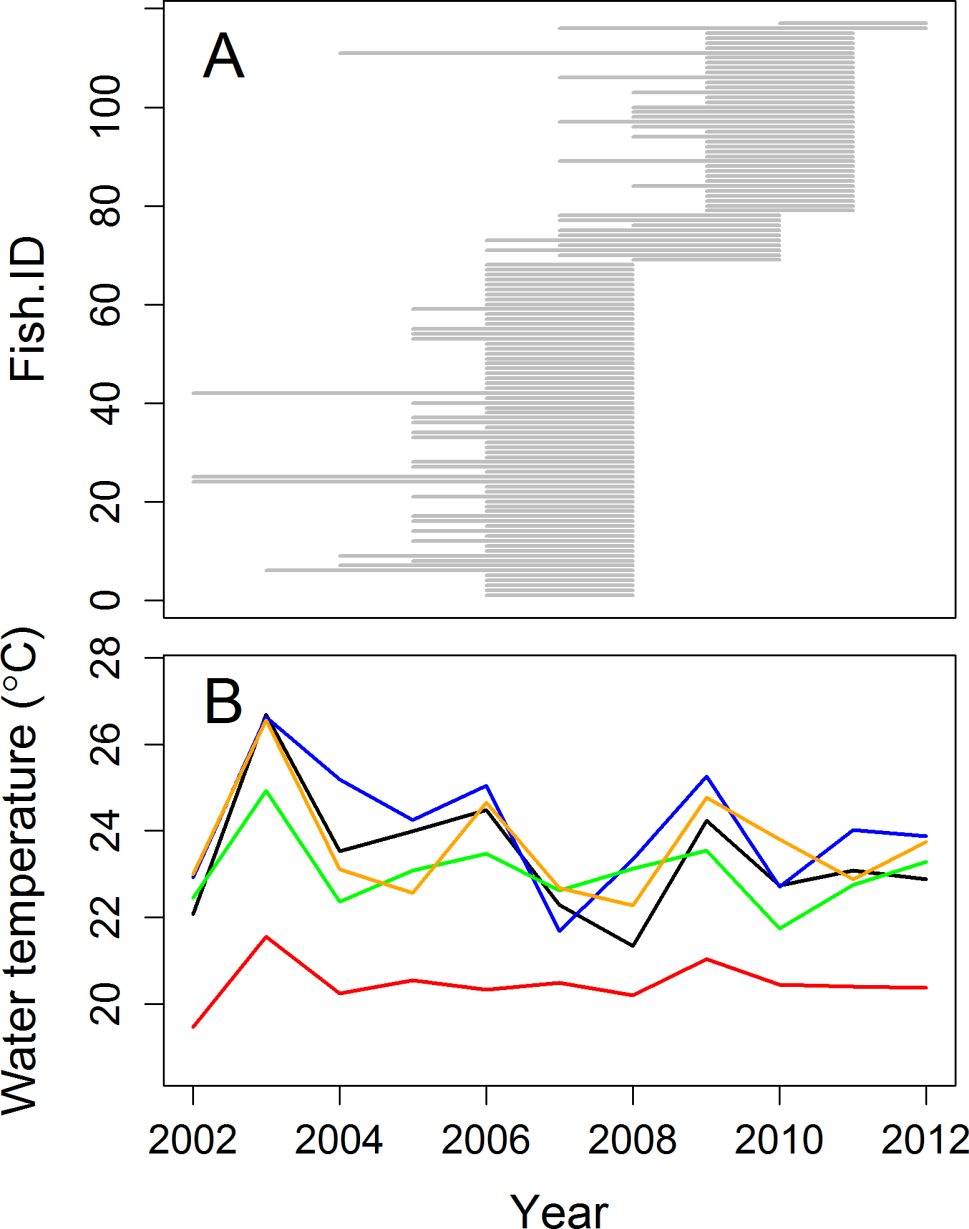
An overview of the data in this study. (**a**) The lifespan of individuals in the study (start of the line represents estimated spawn year and end of the line represents catch year). (**b**) The mean summer temperature values observed at 0-30m depth in the Gulf of Lions (red line) and in the Salses (green line), Bages (black line), Thau (orange line) and Maugio (blue line) lagoons.

For this work, we took advantage of the large number of otoliths (n = 412) already collected for local investigations on the ecology of *S*. *aurata* in the Gulf of Lions [4, 5, 34, 37] (**Fig 2A**). Individuals were caught throughout 2008–2011 in the four coastal lagoons listed above (Bages, Salses, Thau and Mauguio), as well as within 20km of the coastline of the Gulf of Lions. Capture involved a variety of fishing methods including professional fyke nets, demersal trawls, fishing lines, capéchades (traditional Mediterranean fishing nets) and beach seines. All fish were weighed to the nearest 0.1g and total fish length was measured to the nearest 1mm at catch. Fish heads were then removed and stored at -20°C for later removal of the sagittal otolith. No ethics statement is required for this study; all otolith samples were taken from those collected in previously published studies [4, 5, 34, 37].

### Otolith preparation

Only left sagittal otoliths were used for this study. Once removed, they were cleaned in ultrapure water, dried under a fume hood and embedded in epoxy resin (Araldite 2020). 500μm transverse sections that included the otolith core were made perpendicular to the anteroposterior axis using a precision saw (Buehler Isomet 1000). All otoliths were then polished to expose their core by mounting to a slide with thermoplastic glue (Crystalbond™).

Otoliths were imaged using a stereomicroscope (Olympus SZX12) ranging x12.5–90 magnification. Imaging was achieved by a camera attachment (Jenoptik C5 ProgRes®) linked to computer image capture software (Jenoptik ProgRes® CapturePro 2.5).

### Otolith increment measurements

**Fig 3:**
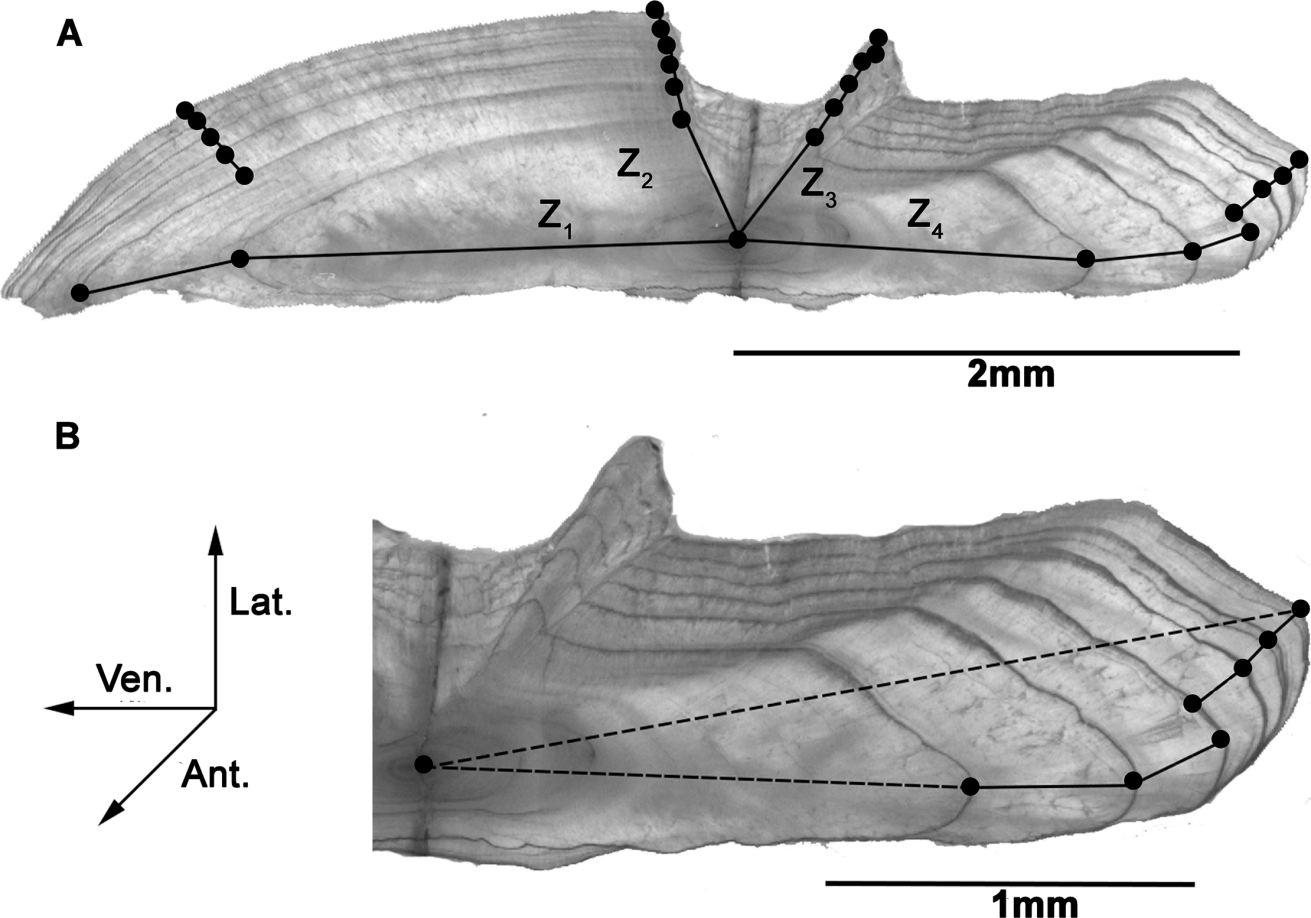
Cross-sections of the left sagittal otolith from a six-year-old *S*. *aurata* individual caught in May 2002. (**a**) Whole sectioned sagittal otolith highlighting the four natural growth axes (Z_1_-Z_4_). (**b**) Magnification of the dorsal region, indicating the contrasting methods of calculating the otolith radius; following the maximal growth axis of each growth increment (sum of solid lines–method used in this study) and the simple measurement from core to the outermost point (dotted line). Orientation: **Ant.**; anterior, **Ven.**, ventral; **Lat.**; lateral axis.

Fish were aged by counting the number of complete opaque bands (annuli) from the core to the outer edge of the otolith. If discrepancies arose about the condition of the edge (translucent or opaque), the month of capture was taken into account, with the assumption that opaque bands formed during the months of December to February [5]. Four radii measures were investigated for each otolith; following the four natural growth axes of the *S*. *aurata* otolith (**Fig 3A**). The first of these follows the maximal ventral growth axis (*Z*_1_ in **Fig 3A**), the second follows the lateroventral axis (*Z*_2_ in **Fig 3A**), the third follows the laterodorsal axis (*Z*_3_ in **Fig 3A**), and the fourth, the maximal dorsal growth axis (*Z*_4_ in **Fig 3A**). Along these four axes, annual increment sizes were measured as the greatest width between two successive annuli (**Fig 3B**). Various landmarks were located on each otolith, including the core (with the aid of a light microscope—Olympus BX41), each annulus (following the maximal growth axis) and the outermost point on each of the four growth axes (see dots on **Fig 3B**). For each of these landmarks, a coloured dot was added to a greyscale image of the corresponding otolith using photo editing software (Adobe Photoshop Elements 9). Increment widths were measured using image analysis and processing software (ImageJ 1.48) and were recorded as a measure of annual otolith growth. Lifetime radii were produced using the summation of the increment widths along each growth axis, including the growth from the outermost annulus to the edge (if applicable). The information gathered, including the images, the four lifetime radii measures, as well as all yearly increment widths, were recorded and added to an existing database comprising the fish capture location, date of capture, weight and total fish length.

The first increment width, from the otolith core to the first annulus refers to the growth in the first 12 months of life (age-0 growth), from the first annulus to second refers to the growth between 12–24 months (age-1 growth) and so forth. As the median spawn date of *S aurata* is generally accepted to be January 1^st^, and the annuli are laid during the winter months (Oct-Mar), each annulus refers to approximately one year of growth. Growth outside of the final annulus (e.g. if an individual was caught in the summer months) was not used in the growth modelling.

We first performed a linear regression analysis on the four radii measures (*Z*_1_ to *Z*_4_ in **Fig 3A**) and total fish length to determine which radius measure was the greatest predictor of total fish length, by which had the greatest R^2^ value, to be used in all further analyses. This along with all other statistical analyses was performed in the statistical program R [38].

### Temperature data

Water temperature records for the Gulf of Lions (43.2–43.7°N, 2.8–5.4°E) between the years 2002 and 2012 were downloaded from www.myocean.eu in the form of ‘reanalysis temperature data’ (**Fig 2B**). This ‘reanalysis’ integrates empirical measurements into a dynamical model with a data assimilation scheme. More specifically, the ‘Nucleus for European Modelling of the Ocean (NEMO)’ was used with the variational data assimilation scheme ‘OceanVAR’. NEMO has been implemented in the Mediterranean at 1/16° by 1/16° horizontal resolution and 72 unevenly spaced vertical levels (Oddo et al., 2009). NEMO is one of the most popular models used within the European Oceanographic community (CMCC, 2014). Only temperature data from the coastal region of the Gulf of Lions was used (latitude>43.2) and at the depths of 1-30m, as this represents the habitat of *S*. *aurata* (FAO, 2005). Daily temperature and oxygen data for the four coastal lagoons (n = 57 buoys situated throughout the lagoons investigated) over the same period (2002–2012) were downloaded from www.ifremer.fr/surval2/ (**S1 Fig**).

Mean summer (June-August) temperatures were preferentially used for this study (**Fig 2B**) as *S*. *aurata*, predominantly those in the northern Mediterranean, experience significantly reduced growth and activity below 13°C [39]. Hence, most of the annual growth occurs in the months of April to October irrespective of fish age and June-August represents the period of maximal growth for *S*. *aurata* juveniles in the lagoons [5]. As the exact summer location of the individuals included in this work (in a lagoon or at sea) was not known, we compared four temperature metrics to assess which was the best predictor of S. aurata annual growth rate in the study population: the summer averages for the coastal region of the Gulf of Lions, for the four lagoons (Mauguio, Bages, Thau and Salses), for the two deep lagoons (Thau and Salses) and for the two shallow lagoons (Mauguio and Bages).

### Growth modelling

To reconstruct the past annual growth rate for all *S*. *aurata* individuals we first modelled the otolith growth, which could then be coupled with an allometry model to estimate fish body growth [29]. To do this we used a series of linear models and linear mixed effects models of varying complexity, both including and excluding intrinsic and extrinsic factors of growth. All of these models were based on the simplest model of using otolith size the previous year *t*-1 (*Z*) to predict the otolith size the following year *t* (*Z’*). This baseline model ignores all extrinsic sources of variation in growth (lm1 in **Table 1**), *Z*′ = *β*_1_*Z* + *α*_0_ + *ε*. As otoliths never fully cease to grow [40], *Z’* will always be greater than *Z*. This type of size-size approach to growth modelling [41, 42] has been shown to accurately capture growth patterns in many animal species [43], including fishes [44]. It is possible to infer from this model the maximal size of the otolith by the point at which the otolith theoretically ceases to grow (*Z* = *Z*′ in the model). The allometric relationship between otolith size and total fish length as well as otolith size and total fish weight were fitted to two linear models (using generalised least squares with exponential variance and ordinary least squares, respectively) to determine whether otolith radius was a better predictor of total fish length or weight. A second pair of linear regression models were also fitted to the log-log relationship between total fish weight and age (in months), as well as the log-log relationship between total fish length and age (in months).

**Table 1:** Summary of the models investigated in this study. The table shows the two linear growth models (lm1-lm2), a linear growth model to estimate initial otolith size given temperature (lm3), a linear allometry model (lm4) and three linear mixed effects growth models (mod1-mod3) used in this study. R^2^ was used to describe the amount of variation in the response variable explained for each of the two simple linear models, whilst marginal R^2^ (mR^2^) values were calculated for the mixed effects models. ΔAIC values were calculated as the absolute difference from mod3; the model with the lowest AIC value.

Model	Equation	R^2^	mR^2^	AIC	ΔAIC
lm1	*Z*′ = *β*_1_*Z* + *α*_0_ + *ε* *Z’* = otolith size at *t*, *Z* = otolith size at *t*-1, ε = error term	0.89	-	-422.1	18.76
lm2	*Z*′ = *β*_1_*Z* + *β*_2_*T* + *α*_0_ + *ε* T = water temperature	0.89	-	-435.1	5.83
lm3	*Z* = *β*_1_*T* + *α*_0_ + *ε*				
lm4	*L* = *β*_1_*Z* + *α*_0_ + *ε* L = total fish length				
mod1	${Z\prime }_{ij}=\beta_{1}Z_{ij}+\alpha_{0}+\alpha_{k}^{t}+\varepsilon_{ij}$ $\alpha_{k}^{t}$ = random effect of k^th^ year, *Z*_*ij*_ = otolith size at t, for i^th^ otolith and j^th^ increment	-	0.89	-426.2	14.69
mod2	${Z\prime }_{ij}=\beta_{1}Z_{ij}+\alpha_{0}+\alpha_{k}^{t}+\alpha_{i}^{F}+\varepsilon_{ij}$ $\alpha_{i}^{F}$ = random effect of i^th^ fish	-	0.88	-434.5	6.38
**mod3**	${Z\prime }_{ij}=\beta_{1}Z_{ij}+\beta_{1}T_{t}^{M}+\alpha_{0}+\alpha_{k}^{t}+\alpha_{i}^{F}+\varepsilon_{ij}$ $T_{k}^{M}$ **= Mediterranean temperature effect**	**-**	**0.89**	**-440.9**	**0**

The von Bertalanffy growth coefficient, *K*, was estimated from the model by: $K=-\log\left(\frac{dZ\prime }{dZ}\right)$, whilst growth performance was calculated using the widely applied phi-prime test; ɸ′ = *ln*(*K*) + 2*ln*(*L*_∞_) [45]. This test gives an indication of the reliability of the von Bertalanffy growth parameters by allowing the comparison the calculated Φ’ with those from other studies; where Φ’ has suggested to be similar between species and genera [46].

We then fitted a series of six increasingly complex mixed effects models to account for intrinsic (e.g. random individual effects) and extrinsic (e.g. water temperature, random year effects) effects on the rate of otolith growth (mod1-mod3 in **Table 1**, mod4-mod6 in **S1 Table**). The simplest of the linear mixed effects models (mod1) included only the year in which the increment formed (t) as a random effect ($\alpha_{k}^{t}$). The second model (mod2) further accounted for individual effects by including the fish ID as a random effect ($\alpha_{i}^{F}$) to the mixed effects model. The remaining four models (mod3-mod6) each included one of four different water temperature variables experienced during the year of increment formation (t) as a fixed effect of growth: mean summer (Jun-Aug) water temperature of the coastal region of the Gulf of Lions (mod3: Mediterranean Temperature $T_{t}^{M}$), mean summer water temperature for the four lagoons (mod4: Lagoon Temperature: $T_{t}^{L}$), mean summer water temperature for the two shallow lagoons, Mauguio and Bages (mod5: Shallow Lagoon Temperature: $T_{t}^{S}$), and mean summer water temperature for the two deeper lagoons, Thau and Salses (mod6: Deep Lagoon Temperature: $T_{t}^{D}$).

A final simplistic linear growth model incorporating temperature (lm2) was developed to be used in a cohort integral projection model, projecting fish growth under different temperature scenarios given the mean otolith size at their first ‘birthday’ (age-0 growth).

The Akaike information criterion (AIC) value was calculated for each linear mixed effect (mod1-mod6) and simple linear (lm1, lm2) growth models to evaluate their predictive performance. Marginal R^2^ was used to test the variance explained by the mixed effects models [47], whilst the R^2^ metric was used for the two simple linear growth models. All models were fitted using the *lme4* package in R [48].

Projected growth curves were estimated using a cohort integral projection model (cIPM) [49],

$$n(Z\prime ,t+1)=\int_{Z_{min}}^{Z_{max}}g(Z\prime |Z)n(Z,t)dZ$$ (1)

where *n*(*Z*,*t*) is the density function of otolith sizes at age *t* and *g*(*Z*′|*Z*) is the growth kernel that projects the otolith size distribution to the next age class. These otolith size distributions for each age class were then used to predict the total fish length distribution using a second integral model,

$$n(L)=\int_{Z_{min}}^{Z_{max}}s(L|Z)n(Z)dZ$$ (2)

where *n(Z)* is the otolith size distribution and *s(L|Z)* is the size kernel, lm4; *L* = *β*_1_*Z* + *α*_0_ + *ε*. Since the model does not include any mortality, the resulting size distributions are conditional on individuals surviving to each age class. We also fitted three linear regression models to parameterise this projection model. The first of which, lm3; *Z* = *β*_2_*T* + *α*_0_ + *ε*, was used to estimate the initial size distribution of the otoliths at their first birthday (age-0 growth) for a given temperature. The second was the growth model (growth kernel), lm2; Z′ = *β*_1_*Z* + *β*_2_*T* + *α*_0_ + *ε*, that was used to estimate the otolith size distributions for the following years (age-1 growth to age-5 growth) given the otolith size distribution for age-0 growth. We performed a bootstrap with 10000 bootstrapped samples to estimate the parameter uncertainty for each of the three models; lm2-lm4. For each bootstrapped sample, an initial otolith size distribution was calculated using lm3 and mean summer temperature (20.48°C) was used as the reference temperature. The following otolith size distributions for each age class were then calculated using Eq 1 (cIPM). Lastly the total fish length distributions were calculated using Eq 2.

The mean, standard deviation, 95% confidence interval of the mean and 95% prediction interval for each of these size distributions were calculated for each age group. The projection was then repeated with reference temperature + 1°C and—1°C.

## Results

The dorsal radius measure (*Z*_4_ in **Fig 3A**, now simply referred to as *Z*) was the best predictor of total fish length (linear regression with ordinary least squares; p < 0.001, R^2^ = 0.809). This measure of otolith size-at-age was therefore used in all further analyses.

### Growth modelling

**Fig 4:**
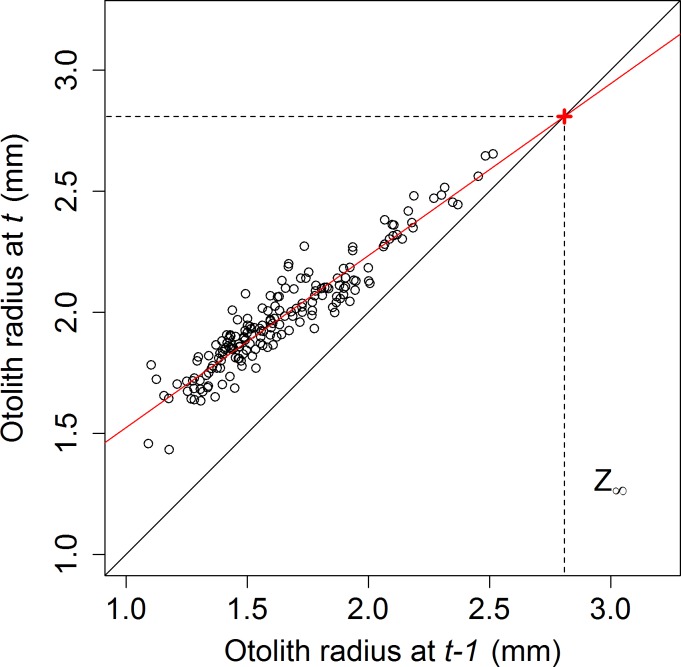
Linear growth of otolith in *S*. *aurata*, modelled as the otolith radius at a given year (*Z*’) against the otolith radius the previous year (*Z*). A linear regression model, *Z*′ = 0.7097 *Z* + 0.8154 + ε, (solid red line) was fitted to this data. The point at which the growth model and the 1:1 line (solid black line) intersect (red cross), represents the theoretical maximal otolith size attainable (dotted black line).

A positive linear relationship was observed between the otolith radius in year *t* (Z’) and otolith radius in the previous year *t-1* (*Z*) (**Fig 4**) for all individuals with at least two complete annuli (age 2+ individuals: n = 116, with a total of 180 increments) (linear regression: *Z*′ = 0.710 *Z* + 0.815 + ε, df = 178, p < 0.001) (lm1 in **Table 1**, also see **Fig 4**). The von Bertalanffy growth parameter *K* was estimated from the negative of the natural log of the slope of the fitted linear regression to be 0.343, and growth performance (phi-prime test) was calculated as 6.42.

**Fig 5:**
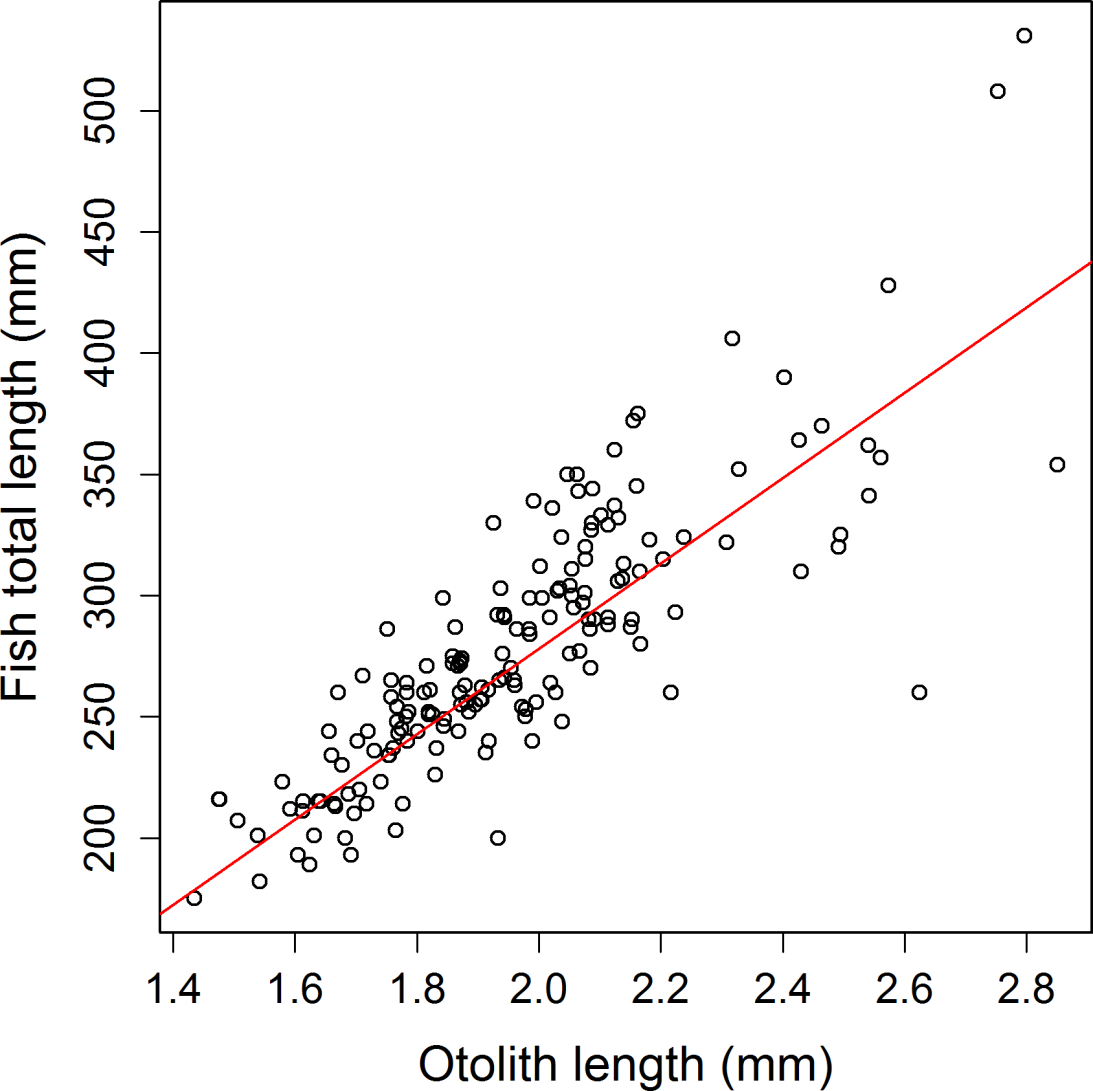
The allometric relationship between total fish length (*L*) and the dorsal radius measure of the sagittal otolith in *S*. *aurata* (n = 412). Fitted line (solid red line). Fitted linear model: *L* = 176.41 *Z*– 74.1. (p < 0.001).

Otolith size (*Z*) was found to be a better predictor of total fish length (*L*) (lm4: linear regression with exponential variance, df = 403, R^2^ = 0.89, p < 0.001, *L* = 176.1 *Z* – 74.1 + ε, Var(ε) = *e*^0.64 *L*^, **Fig 5**) than of total fish weight (W) (linear regression, df = 402, R^2^ = 0.85, p<0.001). Furthermore, age (in months) was found to be a better predictor of total fish length (log-log linear regression, df = 397, R^2^ = 0.81, p<0.001) than total fish weight (log-log linear regression, df = 396, R^2^ = 0.78, p<0.001, **S1 Fig**). Further analyses therefore focussed on the growth of total fish length rather than of total fish weight. Applying lm4, the theoretical maximal size attainable (*L*_*∞*_) of *S*. *aurata* was estimated to be 420mm, given an estimated maximal otolith size (*Z*_*∞*_) of 2.81mm (see ‘+’ symbol in **Fig 4**).

Of the six mixed effects growth models, mod3 had the greatest predictive performance. This model included both *fish ID* and *Year* as random effects and otolith size at previous year (*Z*) and *mean Mediterranean summer temperature* (*T*^*M*^) as fixed effects, and was used as the baseline for AIC comparison (ΔAIC = 0). The next best model, mod2, did not include a temperature variable as a fixed effect but included *fish ID* and *Year* as random effects (ΔAIC = 6.38). Mod4, mod5 and mod6 with *mean lagoon summer temperature* (*T*^*L*^), *mean shallow lagoon summer temperature* (*T*^*S*^) and *mean deep lagoon summer temperature* (*T*^*D*^) as fixed effects respectively, all had similar ΔAIC values; 7.64, 7.68 and 7.73, respectively. Whilst, mod1 was the poorest model (ΔAIC = 14.69). All models had similar R^2^ and marginal R^2^ values, ranging 87.6% to 89.0% (**Table 1**).

**Fig 6:**
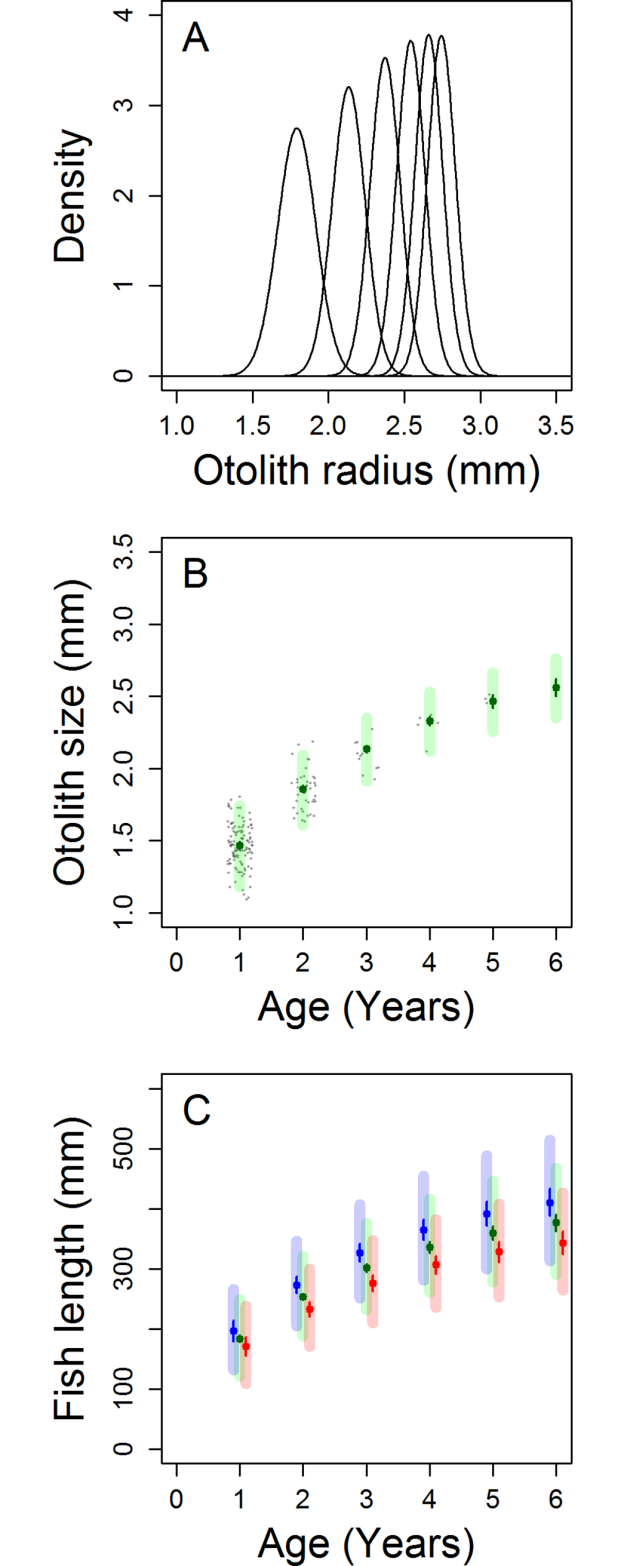
Visualisation of the method used to project from initial otolith size to total fish length at each age class. (**a**) Density plot of otolith size at each age group; the leftmost distribution refers to the initial otolith size (age-0 growth) and the following distributions to age-1 to age-5 growth. (**b**) Otolith size for each age group. Means and confidence intervals were derived from the density plots. The observed data (grey dots) have been ‘jittered’ to help visualise the scatter of the data at each age class. (**c**) Projected total fish length at age under three temperature scenarios: mean summer temperature of the Gulf of Lions over the study period (green), a one degree cooler system (blue) and a one degree warmer system (red). Solid lines represent the 95% confidence intervals for the mean total fish length, whilst the thicker translucent lines represent the 95% prediction interval. The total fish size was derived from the allometric relationship between otolith size and total fish size. Confidence and prediction intervals in (b) and (c) were derived from bootstrapped samples.

The initial otolith size distribution (age-0 growth) was estimated from a linear regression model (lm3 in **Table 1**) of summer temperature and otolith size (*Z*) (see leftmost normal-distribution in **Fig 6A** and age-0 data point in **Fig 6B**). The mean fitted model over all bootstrapped samples was: *Z* = 2.937 − 0.0718 *T*^*M*^; predicting increased summer temperatures to have a negative effect of on *S*. *aurata* growth. On the other hand, no relationship was observed between summer oxygen levels and *S*. *aurata* growth (linear regression model: df = 115, p>0.5). The simplest linear growth model incorporating temperature (lm2) was fitted to *Z* against *Z*’ for each bootstrapped sample with mean parameter estimates of; *Z*′ = 0.7033 Z − 0.0631 T + 2.120. Lm2 was then used to estimate the otolith size distributions for the following age groups (age-1 to age-7: see normal distribution curves 2–8 in **Fig 6A** and age-1 to age-7 data points in **Fig 6B**). Finally, the allometry model (lm4) previously used, was fitted to the bootstrapped samples with mean parameter estimates of: *L* = 176.38 *Z* − 74.62, and was used to estimate the size distribution of total fish length for each age class (**Fig 6C**). The projection was repeated using 21.48°C (1°C increase from the reference temperature–see red lines in **Fig 6C**) and 19.48°C (1°C decrease–see blue lines in **Fig 6C**).

The cIPM predicts that a two year old individual would be expected, on average, to be 7.9% smaller with a 1°C increase in mean summer water temperature (233mm), as compared to current temperatures (253mm). Equally a three year old individual would be expected, on average, to be 8.6% smaller (276mm) than it is currently (302mm).

## Discussion

With many fish stocks around the globe already fully exploited and a growing reliance on aquaculture for fish production [50] accurate knowledge of how environmental change is impacting fish life-histories is needed to inform ecosystem-based fisheries management and vulnerability assessments under climate change [9]. Fisheries stock assessments that combine growth rate, mortality and recruitment to model population dynamics and assess exploitation status, rarely have enough information to take into account the effects of environmental change [51] and often rely on cross-sectional data or snapshots of size-at-age [52]. Basing growth rate predictions solely on cross-sectional data relies on the assumption that factors affecting growth are constant through time, and may result in inaccurate growth estimations. In contrast, using longitudinal data permits growth to be measured at the individual level, providing a more robust method of growth calculation. Furthermore, it is important to develop a wide range of growth models as they each provide their own limitations and advantages, and allow for multi-model inferences, which can provide better estimates than only implementing the von Bertalanffy growth function [5, 53].

Increased water temperature has been shown to result in contradictory effects of increased enzymatic activity [54] and reduced oxygen availability; the balance of which determine the directionality of the effect on growth [55]. Understanding this relationship in a number of interacting species is key to being able to accurately predict fish population dynamics with increasing water temperatures. The temperature-size rule for ectotherms proposed by Atkinson [55] states that individuals tend to mature earlier and therefore have reduced adult size with higher temperatures [19, 21]. *S*. *aurata* are able to tolerate temperatures up to 26–30°C, although they grow optimally at 25°C [56, 57]. An increase of 3.0°C in mean global sea surface temperature, as predicted by the end of the century if we continue business as usual (between RCP6.0 and RCP8.5) [9] would take maxima water temperature in the Gulf of Lions to 29.7°C, which is approaching this maximal tolerable temperature for this species.

The current study is the first example of an observational study showing a negative effect of temperature on L_∞_ in wild *S*. *aurata* populations. This apparent decline in growth of *S*. *aurata* at higher summer temperatures is likely due to reduction in growth at temperatures above 25°C, the optimal rearing temperature for *S*. *aurata* [57, 58]. It is possible for lowered oxygen in certain habitats during the summer month to also impact *S*. *aurata* growth. The limiting oxygen saturation (LOS) is the oxygen threshold required to maintain routine metabolic rates [24]. As oxygen levels approach this threshold, *S*. *aurata* utilise regulatory mechanisms such as increased gill ventilation and perfusion [59]. Below the LOS threshold, fish must increase their anaerobic respiration, a more inefficient method of ATP synthesis [60]. The LOS threshold is dependent on both the species and the water temperature, known to impact fish metabolic rate [24, 61]. The lowest mean summer oxygen concentration observed in the lagoons (5.9 mg O_2_ L^-1^ in Thau, 2006 –see **S2 Fig**), is significantly greater than the limiting oxygen saturation (LOS) for this species (~2.3 mg O_2_ L^-1^ at 19°C) [24, 62]. It is therefore is unlikely that the *S*. *aurata* populations in the lagoon are subject to chronic hypoxic conditions. However, daily oxygen concentrations were found to drop as low as 0.1 mg O_2_ L^-1^ (Thau 24/07/06, Mauguio 17/06/03 and 20/07/06) following heatwave events [22]. This hypoxia in the warmer shallow lagoons may not have a direct impact on *S*. *aurata* growth as individuals can actively avoid these areas to reduce hypoxic stress, however, there may be indirect effects on *S*. *aurata* growth via benthic prey mortality [22, 25, 26].

As reported by Walford [42], plotting size at year *t*, against size at the previous year *t-1*, to model growth, often creates a linear pattern [63]; as was seen in this study. Creating a linear function of growth, as opposed to the asymptotic von Bertalanffy growth function, simplifies the modelling process and comparison with other studies.

The cross-sectional analysis (using only size and age at capture information) ignores important life-history information such as the growth trajectory experienced by an individual to achieve its current size. For example, if an individual experienced exceptional growth during one year, it may remain as a consistently larger individual relative to its cohort, biasing the size-at-age distribution based on cross-sectional data. This does not occur in a size-size based approach using longitudinal data, as growth is based on size changes within each year of life. Therefore, a year of exceptional growth would be represented as a single point deviation from the linear modelled growth rate and thus allow the identification of causal extrinsic factors such as temperature. Methods based on cross-sectional data will always give a biased perspective on growth unless size-selective mortality can be accounted for. Longitudinal data, as used here, will yield unbiased estimates of growth when among-individual variation is negligible.

The linear growth model applied here estimated growth performance (phi-prime) (6.42) within the range of previous estimates using cross-sectional data in *S*. *aurata* from the Gulf of Lions (6.34–6.81) [5]. This study using individual-level growth, however, estimated a much lower mean *L*_*∞*_ value and greater mean *K* value for 2002–2012 (*L*_*∞*_ = 420mm, *K* = 0.35yr^-1^) than previous studies (*L*_*∞*_ = 577 – 723mm, *K* = 0.11–0.27) [5]. Given the data collected here, we would expect a six year old individual sampled in the years 2002–2012 to be between 30.5cm and 49.5cm (95% prediction interval). It is important to note however that most of the individuals in this study were below four years of age and <40cm total fish length. The *L*_*∞*_ reported here is unlikely to accurately represent the real-life maximal size as *S*. *aurata* individuals greater than 42cm are regularly caught in this region of the Mediterranean [64]. This value, however, corresponds to the mean value of the size distribution at each age group. Given more data of older individuals, it would be possible to extend this model to incorporate older age groups and provide a better estimate of the total fish length maxima.

The cIPM used in this study allowed the incorporation of temperature into modelling growth rate; equally this could be extended to use any other environmental variables (e.g. salinity, rainfall or wind speed). Temperature is one of the most influential factors affecting the growth rate of fish [65], directly impacting the availability of food [66], metabolic rate [67] and dissolved oxygen content of water [68]. Predicting the effect of temperature on growth rate is vitally important as growth rate influences other physiological factors such as size-at-age, size at sexual maturity and maximal size. Likewise, the size of individuals has impacts on their reproductive output [69], their interaction within the food web [70], as well as their commercial value. Intensive farming of *S*. *aurata* occurs mostly in coastal sea cages [71], thus changes in sea temperature impacts both wild and aquaculture *S*. *aurata* populations. Furthermore, in the warmer waters of the southern Mediterranean, summer temperatures are likely to be greater than the upper tolerable limit of *S*. *aurata*, thus possibly resulting in severe consequences for wild *S*. *aurata* in these regions. The effect of temperature on growth rate is of economic concern to intensive *S*. *aurata* farms, as slower growth could result in the time to reach a commercial size (350-400g), roughly one year in optimal conditions [2], to be increased. In fisheries management, stock assessments rely on accurate size-age information as well as the age-structure of the stock, age at first spawning and growth rate. Therefore, decreasing mean total fish length as a result of increasing water temperature could result in skewed stock estimates and inaccurate quotas.

### Model limitations

Whilst this study was able to use increment widths to determine growth within each year of life, the exact location of each individual within each growth season, either in a lagoon or in the Gulf of Lions, was unknown. Hence, the exact temperature experienced by each individual within each year was also unknown. This study, therefore, used four temperature variables (mean summer temperatures for the Gulf of Lions, four lagoons, shallow lagoons, and deep lagoons); of which including the Gulf of Lion's temperature (T^M^); providing the best fitting model. To overcome this issue of unknown temperature experienced, it would be beneficial to be able to predict the location of individuals within the summer months. This could be achieved either by microchemical analysis [3] or the incorporation of a hidden Markov model [72]. A hidden Markov model would allow the prediction of the likelihood of an individual occurring in each of the lagoons or the Gulf of Lions, based on the estimated relationship between temperature and growth. This would produce a more robust analysis of the effect of temperature on individual-level growth.

This model could easily be applied to, and may even be more fitting to, an aquaculture system, whereby the environmental conditions for each individual and the individuals themselves can be directly measured, overcoming the major limitation of temperature estimation and indirect size measurements. Finally, the use of longitudinal data assumes juvenile growth of older individuals is representative of the juvenile growth of the true population at the earlier stage, which may introduce bias. To overcome this, further development of the cIPM outlined here could include size-dependent mortality terms. Nonetheless, this study provides a methodological basis for the use of longitudinal data in the prediction of environmental effects on *S*. *aurata* growth rate and may form a basis for future studies.

## Conclusions

The analysis of 11 years of detailed coastal water temperature records and fish historical annual growth rates (from over 400 juvenile and mature individuals) suggested that, with mean summer water temperatures increasing in the Gulf of Lions, local *S*. *aurata* has already started decreasing in size at a given age. The reduced growth rate with increasing water temperatures observed so far could be due to summer water temperatures being greater than the optimal rearing temperature of this species, as well as potential indirect effects on growth by reduced food availability due to prey mortality in the shallow coastal lagoons where *S*. *aurata* inhabit. This phenomenon could be further intensified by water temperatures nearing that of the upper tolerable limit for this species; predicted by the end of the century if emission-mitigation measures are not applied. The methodology outlined in this study, whilst here applied to wild *S*. *aurata* populations, could be better applied to an aquaculture setting where detailed individual-level environmental information is known.

## Supporting information

S1 Fig Linear relationship between the logged total fish weight (g) and the logged age (in months) in Sparus aurata. The linear model was calculated to be log(weight) = 1.18 + 1.32*(log(age)), where weight in measured in grams and age in months. Otolith radius refers to the dorsal axis radius measure of the otolith (Z_4_ in Fig 3). R^2^ = 0.78, p < 0.001.
(TIFF)

S2 Fig Dissolved oxygen concentrations and surface water temperatures of the four major coastal lagoons in the Gulf of Lions; Salses-Leucate (Salses), Bages-Sigean (Bages), Thau and Mauguio, for the years 2002–2012. (TIFF)

S1 Table Four linear mixed effects models, each with a differing temperature variable. (DOCX)
