# Supplementary figures and images for: Using an integral projection model to assess the effect of temperature on the growth of gilthead seabream Sparus aurata
Source: PLoS One. 2018 May 3;13(5):e0196092. doi: 10.1371/journal.pone.0196092 (PMC5933764; doi:10.1371/journal.pone.0196092)

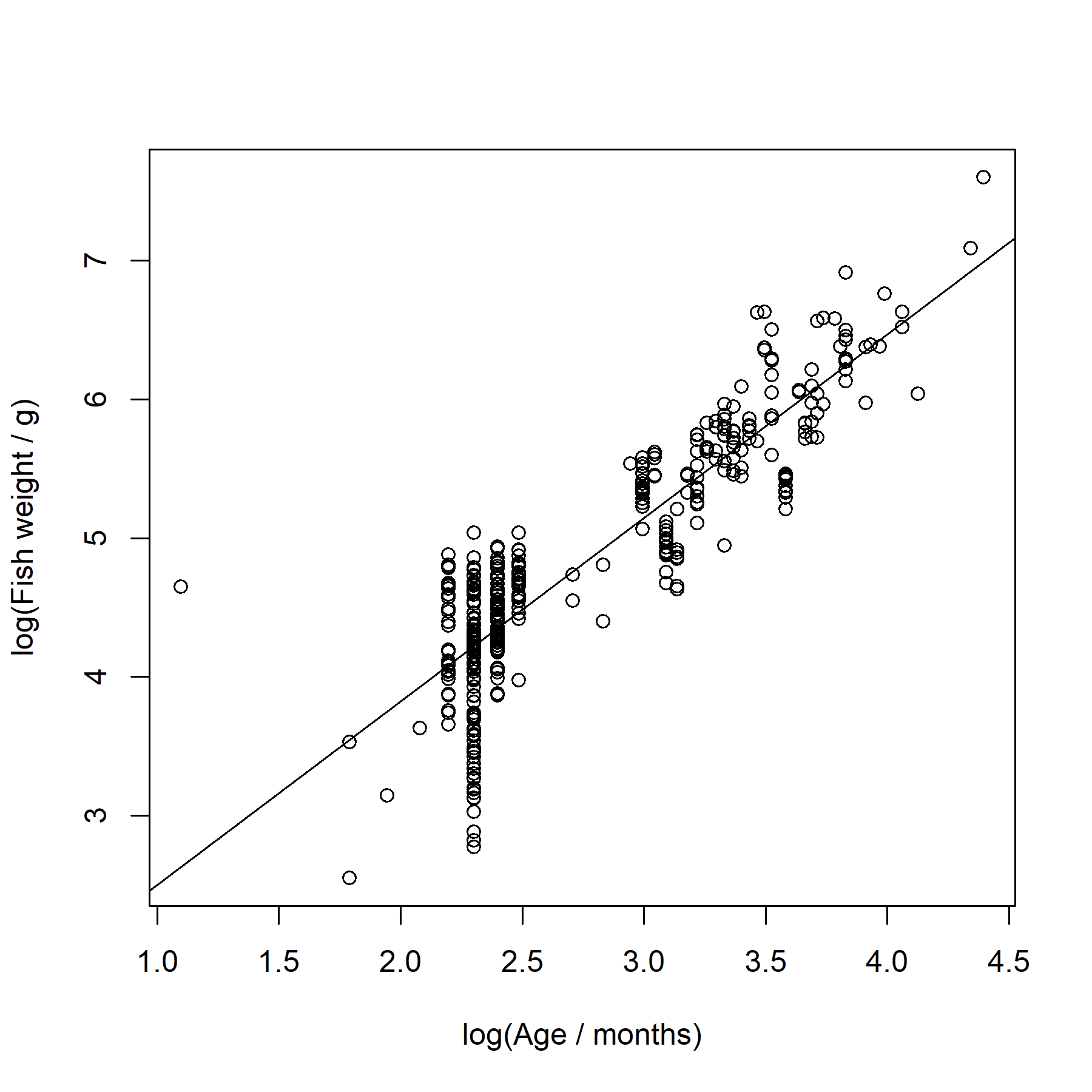

Supplement: S1 Fig — The linear model was calculated to be log(weight) = 1.18 + 1.32*(log(age)), where weight in measured in grams and age in months. Otolith radius refers to the dorsal axis radius measure of the otolith (Z4 in Fig 3). R2 = 0.78, p < 0.001. (TIFF) [file pone.0196092.s001.tiff]

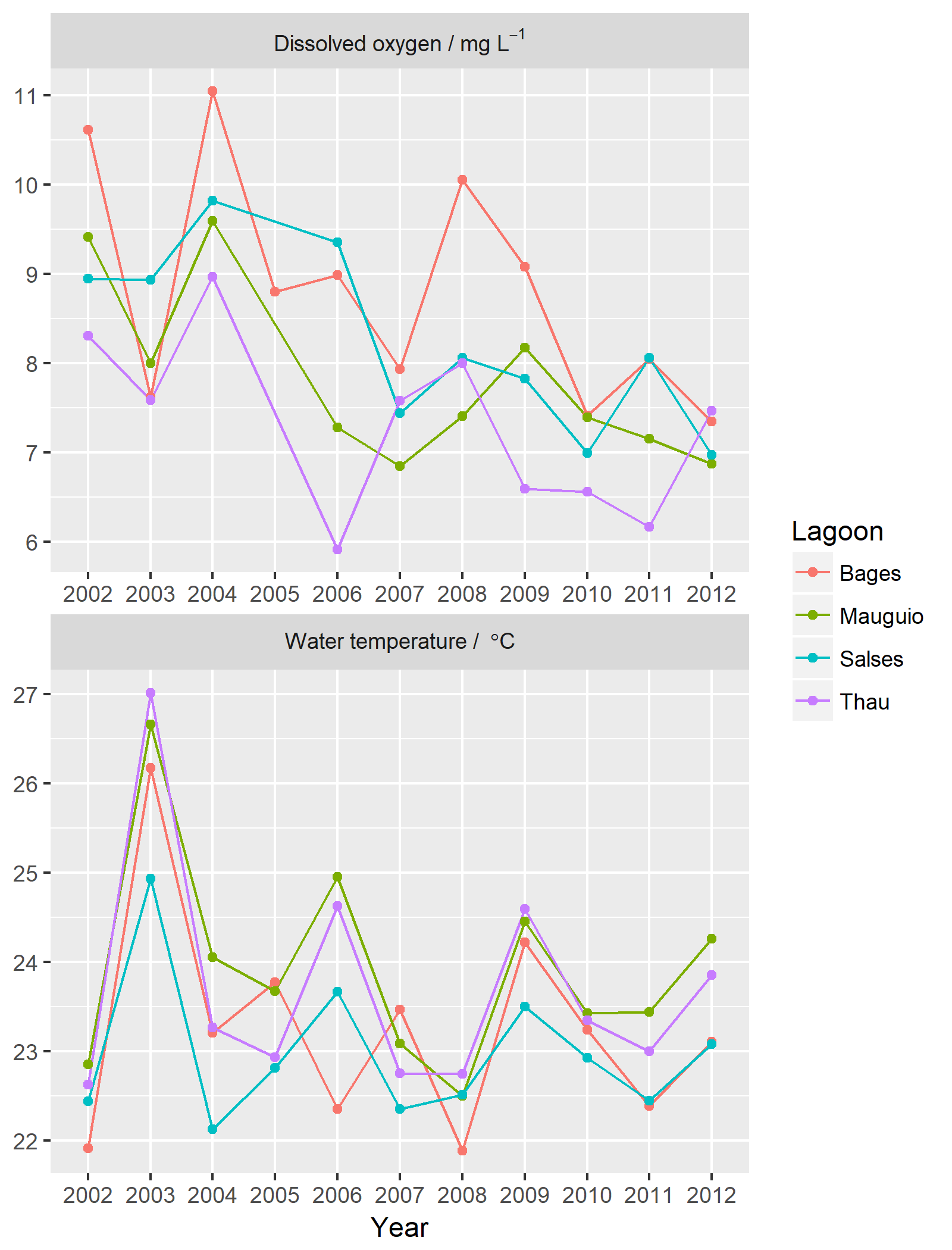

Supplement: S2 Fig — (TIFF) [file pone.0196092.s002.tiff]
