# Supplementary material for: Using an integral projection model to assess the effect of temperature on the growth of gilthead seabream Sparus aurata
Source: PLoS One. 2018 May 3;13(5):e0196092. doi: 10.1371/journal.pone.0196092 (PMC5933764; doi:10.1371/journal.pone.0196092)
Supplement: S1 Table — (DOCX) [file pone.0196092.s003.docx]

| Model | Equation | R^2^ | mR^2^ | AIC | ΔAIC |
| --- | --- | --- | --- | --- | --- |
| **mod3** | $\boldsymbol{Z'}_{\boldsymbol{ij}}\boldsymbol{=}\boldsymbol{\beta}_{\boldsymbol{1}}\boldsymbol{Z}_{\boldsymbol{ij}}\boldsymbol{+}\boldsymbol{\beta}_{\boldsymbol{1}}\boldsymbol{T}_{\boldsymbol{t}}^{\boldsymbol{M}}\boldsymbol{+}\boldsymbol{\alpha}_{\boldsymbol{0}}\boldsymbol{+}\boldsymbol{\alpha}_{\boldsymbol{k}}^{\boldsymbol{t}}\boldsymbol{+}\boldsymbol{\alpha}_{\boldsymbol{i}}^{\boldsymbol{F}}\boldsymbol{+}\boldsymbol{\varepsilon}_{\boldsymbol{ij}}$  $\boldsymbol{T}_{\boldsymbol{k}}^{\boldsymbol{M}}$ **= Mediterranean temperature effect** | **-** | **0.89** | **-440.9** | **0** |
| mod4 | ${Z'}_{ij}= \beta_{1}Z_{ij}+ \beta_{1}T_{t}^{L}+ \alpha_{0}+ \alpha_{k}^{t}+ \alpha_{i}^{F}+ \varepsilon_{ij}$  $T_{k}^{L}$ = lagoon temperature effect | - | 0.88 | -433.3 | 7.64 |
| mod5 | ${Z'}_{ij}= \beta_{1}Z_{ij}+ \beta_{1}T_{t}^{S}+ \alpha_{0}+ \alpha_{k}^{t}+ \alpha_{i}^{F}+ \varepsilon_{ij}$  $T_{k}^{S}$ = Shallow lagoon temperature effect | - | 0.88 | -433.2 | 7.68 |
| mod6 | ${Z'}_{ij}= \beta_{1}Z_{ij}+ \beta_{1}T_{t}^{D}+ \alpha_{0}+ \alpha_{k}^{t}+ \alpha_{i}^{F}+ \varepsilon_{ij}$  $T_{k}^{D}$ = Deep lagoon temperature effect | - | 0.88 | -433.2 | 7.73 |
